# Visualization of DNA Replication in Single Chromosome by Stable Isotope Labeling

**DOI:** 10.1247/csf.21011

**Published:** 2021-09-25

**Authors:** Kosuke Nagata, Ken-ichi Bajo, Hideyuki Mitomo, Ryosuke Fujita, Ryota Uehara, Kuniharu Ijiro, Hisayoshi Yurimoto

**Affiliations:** 1 Natural History Sciences, Hokkaido University, Sapporo 001-0021, Japan; 2 Research Institute for Electronic Science (RIES), Hokkaido University, Sapporo 001-0021, Japan; 3 Global Station for Soft Matter, Global Institution for Collaborative Research and Education, Hokkaido University, Sapporo 001-0021, Japan; 4 Laboratory of Sanitary Entomology, Department of Bioresource Science, Faculty of Agriculture, Kyushu University, Fukuoka 819-0395, Japan; 5 Graduate School of Life Science, Hokkaido University, Sapporo 001-0021, Japan; 6 Faculty of Advanced Life Science, Hokkaido University, Sapporo 001-0021, Japan

**Keywords:** stable isotope, chromosome replication, semi-conservative replication, imaging, mass spectrometry

## Abstract

Among the inheritance of cellular components during cell division, deoxyribonucleic acid (DNA) and its condensate (chromosome) are conventionally visualized using chemical tag-labeled nucleotide analogs. However, associated mutagenesis with nucleotide analogs in the visualization of chromosomes is cause for concern. This study investigated the efficiency of using stable isotope labels in visualizing the replicating cultured human cell-chromosomes, in the absence of analog labels, at a high spatial resolution of 100 nm. The distinct carbon isotope ratio between sister chromatids reflected the semi-conservative replication of individual DNA strands through cell cycles and suggested the renewal of histone molecules in daughter chromosomes. Thus, this study provides a new, powerful approach to trace and visualize cellular components with stable isotope labeling.

## Introduction

In cell division, many molecules and organelles (e.g., chromosomes, mitochondria, and chloroplasts) are inherited from parent cell to daughter cell. Chromosomes (and DNA) have been well studied in its inheritance by visualization, and many techniques and tools have been developed. Labeling and tracing DNA molecules with nucleotide analogs such as bromodeoxyuridine (BrdU) or with radioisotopes have been used (Gratzner *et al.*, 1975; Latt, 1973; Levi-Setti and Le Beau, 1992; Perry and Wolff, 1974; Pinkel *et al.*, 1985; Taylor *et al.*, 1957). Limitations of radioisotope or nucleotide analog-labeling include DNA fragmentation and mutation. (Solary *et al.*, 1992; Taupin, 2007). It has also been reported that radioisotopes and analogs are harmful to cells (Bannigan and Langman, 1979; Lehner *et al.*, 2011; Ross *et al.*, 2008; Solary *et al.*, 1992). In this regard, stable isotope labels are suitable alternates to radioisotopes and analogs in labeling biomolecules. Stable isotopes such as ^13^C, ^15^N, and ^18^O are examples of markers used in secondary ion mass spectrometry (SIMS) with no observable effect on biological metabolism (Hamasaki *et al.*, 2013; Kuga *et al.*, 2014; Lechene *et al.*, 2006; Steinhauser *et al.*, 2012). However, stable isotope imaging is currently limited to tracing cellular-level metabolism (Steinhauser *et al.*, 2012).

In this study, we visualized the distributions of stable isotope labels incorporated into the chromosomes of a human cultured cell at a spatial resolution of 100 nm. Furthermore, we quantified the dynamics of labeled chromosome segregation through successive cell division using stable isotope imaging. This analog label-free imaging system also enabled us to trace protein recycling through cell cycles, paving the way for analyzing diverse characteristics within individual organelles.

## Materials and Methods

### Cell culturing and chromosome labeling

**Fig. 1:**
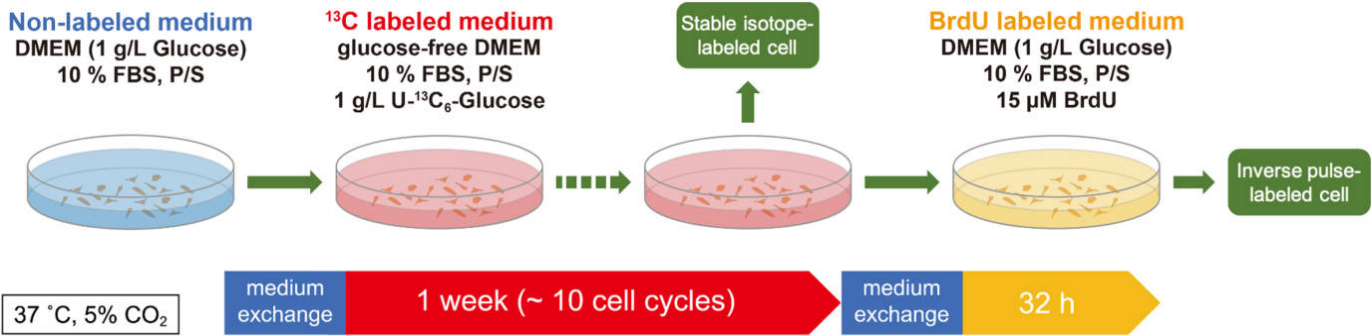
Schematic representation of the U-^13^C_6_-Glucose and BrdU labeling protocol. HeLa cells were cultured in culture media containing 1 mg/mL U-^13^C_6_-Glucose for 1 week. After stable isotope labeling, a portion of the cells was processed for observation. The remaining cells were cultured in culture media, with a natural carbon isotope ratio, laced with BrdU. Cells were processed for observation after a 32-hour incubation period. Cell cultures were washed with PBS before medium replacement.

HeLa cells were cultured in 2 mL of Dulbecco’s modified Eagle’s medium (DMEM) (Sigma) supplemented with 10% Fetal bovine serum (FBS) (Sigma) and penicillin (100 unit/mL)-streptomycin (100 μg/mL) (P/S) (Wako) on 35 mm tissue culture dishes in a humidified 5% CO_2_/95% atmosphere at 37°C (Fig. 1). For ^13^C isotope labeling, cells were washed with PBS (1.0 mL) and incubated with glucose-free DMEM (Wako) supplemented with U-^13^C_6_-Glucose (1 mg/mL) (Cambridge Isotope Laboratories, Inc.) (2.0 mL), 10% FBS (Sigma), and P/S. After three passages in 7 days, cells were divided for chromosome spread preparation and ^13^C-inverse pulse labeling, respectively. In the ^13^C-inverse pulse labeling, cells were washed with PBS (1.0 mL) and incubated with Glucose (1 mg/mL) (natural carbon isotope ratio: 0.011)-containing DMEM (Sigma) (2.0 mL) supplemented with bromodeoxyuridine BrdU (15 μM) (Sigma), 10% FBS and P/S for 32 h. The resulting cells were also used for chromosome spread preparation.

### Chromosome spread preparation

Cells were treated with colcemid (50 ng/mL) (Nacalai Tesque Inc.) for two hours to arrest the cell cycle in mitosis, harvested, and washed with PBS. Cells were treated with hypotonic KCl (75 mM, 8 min) followed by fixative solution (absolute methanol: acetic acid=3:1) that was refreshed every 3 minutes for 15 minutes (Levi-Setti and Le Beau, 1992). A drop of cell suspension was put on a Si wafer (5×5 mm^2^) and air-dried for observation.

### Isotope imaging

A scanning electron microscope (JEOL JSM-7000F) was used in locating chromosome spreads and acquiring secondary electron (SE) images. SE images were acquired with a probe current of 2 nA and an acceleration voltage of 5 keV. Ion-induced SE images were acquired with a secondary neutral mass spectrometer (JEOL LIMAS). Ion-induced SE images were obtained with gallium (Ga) focused ion beam current of 3 pA and an acceleration voltage of 20 kV (Ebata *et al.*, 2012). The secondary neutral mass spectrometer also enabled analysis with a high lateral resolution of up to 10 nm and high mass resolution of 10^6^ for full width half maximum (Bajo *et al.*, 2016, 2019; Ebata *et al.*, 2012; Nagata *et al.*, 2019; Tonotani *et al.*, 2016; Yurimoto *et al.*, 2016). In this regard, the primary ion beam was focused to a diameter of 100 nm at a current of 400 pA with an acceleration voltage of 20 kV (Nagata *et al.*, 2019). The primary beam scanned a 19.2×33.7 μm^2^ area at an incident angle of 35° from the sample surface to acquire a 300×300 pixel ion imaging. Post-ionized ions were accumulated for 500 primary beam pulses at each pixel to collect data.

Prior to chromosome measurements, the surface of the Si wafer substrate was pre-sputtered to remove cell components overlaying the chromosome and the Si wafer substrate (Fig. S1).

Post ionized ^12^C^+^, ^13^C^+^, ^28^Si^2+^, ^79^Br^2+^, and ^81^Br^2+^ ions, that had sputtered from the sample surface were observed. Negligible levels of ^12^CH^+^ interference were observed in the ^13^C^+^ peak from chromosomes. The contribution of ^12^CH^+^ was 3% at the mass resolving power of 6800 (FWHM) (Fig. S2). No interference was detected in the other ion peaks.

### Data analysis

^79^Br^2+^ and ^81^Br^2+^ isotopes were detected and counted for Br ion images. ^13^C and Br counts were normalized by total carbon counts (^12^C+^13^C) to correct for small fluctuations in ion intensities during measurements. A smoothing filter of 3×3 pixels was applied to isotope images to reduce statistical noise. The intensity of ^28^Si^2+^ masked signals outside the chromosomes generated from the Si substrate (Fig. S3).

## Results

**Fig. 2:**
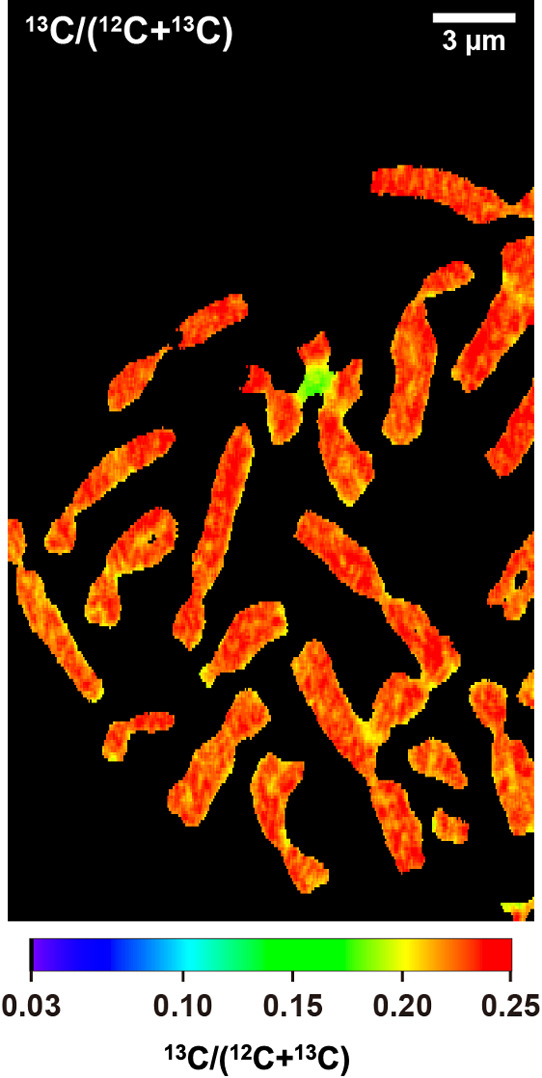
Carbon isotope ratio image of a chromosome spread from a single HeLa cell cultured in media laced with a stable isotope, for 1 week. The color bar indicated the carbon isotope ratio [^13^C/(^12^C+^13^C)]. The scale bar was also indicated.

To label cellular molecules, we cultured HeLa cells with U-^13^C_6_-Glucose-containing medium (Fig. 1). Chromosomes were spread on the silicon wafer and detected under SEM. The ion images (^12^C and ^13^C) for ^13^C-labeled chromosomes were obtained in LIMAS analysis (Fig. 2). The average resultant isotope ratio [^13^C/(^12^C+^13^C)] of the chromosomes was 0.230 with a standard deviation of 0.011. This value was significantly higher than the natural carbon isotope ratio (0.011) but on par with the U-^13^C_6_-Glucose-treated culture medium (0.238). This observation confirmed the successful and efficient labeling of the chromosomes.

**Fig. 3:**
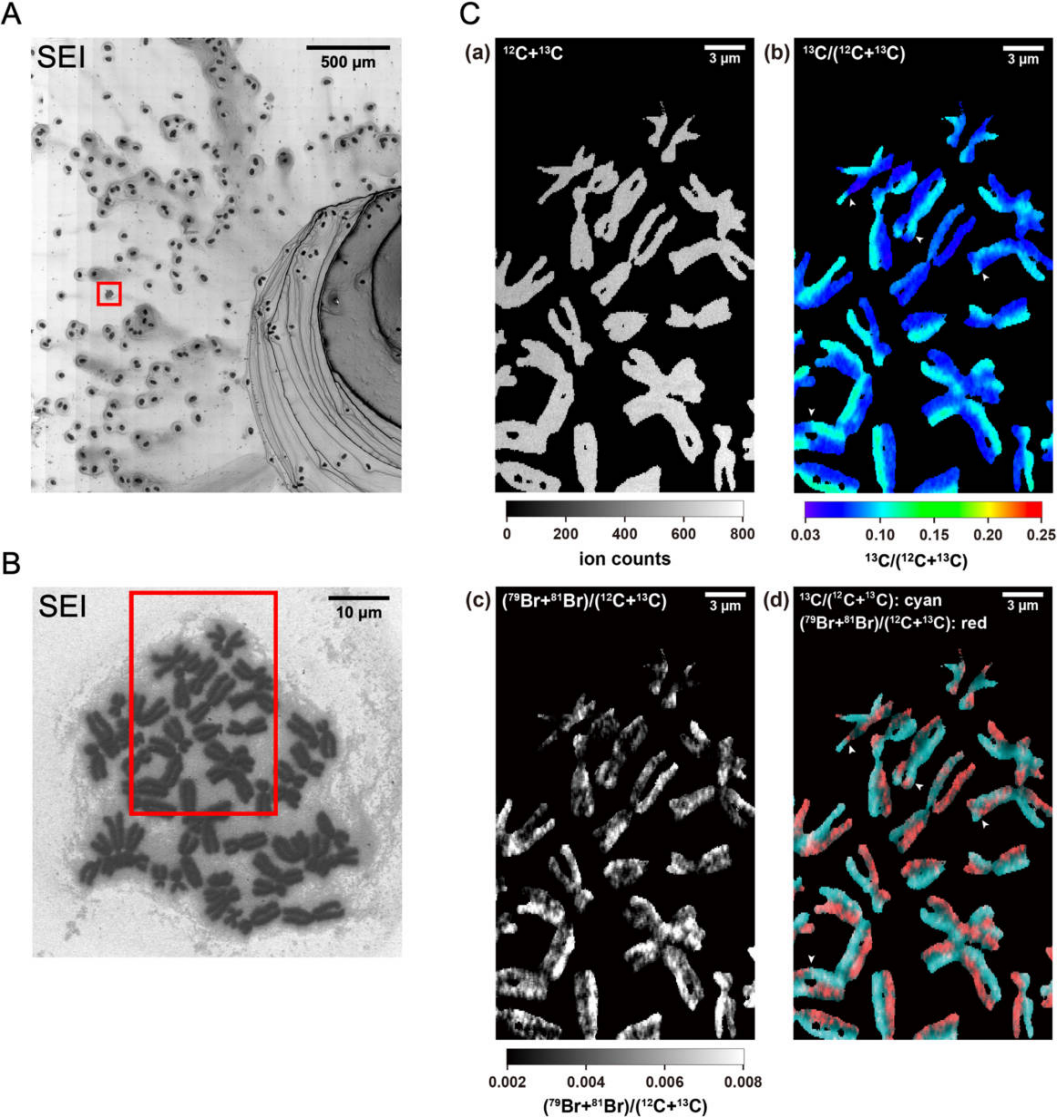
(A) Secondary electron image of chromosome spreads mounted on an Si wafer substrate. (B) Secondary electron image of a chromosome spread of one HeLa cell. The interspace between each chromosome is filled with cell components. The red square indicates the area scanned to obtain the ion image shown in panel C and Fig. S3. (C) Images of a chromosome spread from a single HeLa cell incubated with DMEM laced with 15 μM BrdU for 32 h after stable isotope labeling. (a) ^12^C+^13^C. (b) Carbon isotope ratio. (c) (^79^Br+^81^Br)/(^12^C+^13^C). (d) Merged image of (b) (cyan) and (c) (red). White arrowheads indicate the position of sister chromatid exchanges (SCEs).

The U-^13^C_6_-Glucose-treated culture medium was then replaced with a ^13^C-free medium to inverse-label the chromosomes. We also added BrdU to track the progression of ^13^C-labeled chromosomes through the cell cycle(s). The majority of cells were in the mitotic phase marked by the presence of condensed chromatids (Fig. 3). The cells were considered to have gone through two cycles of cell division due to the heterogeneous distribution of ^13^C and Br between sister chromatids. In the cell spreads observed under SEM imaging (Fig. 3A), we initially determined the distribution of ^13^C-labeled chromosomes in 8 individual cells, which were labeled utilizing identical protocol. All eight analyzed cells showed the same ^13^C distribution pattern (Fig. S4), and one of them was further analyzed for fine imaging (Fig. 3B and 3C). Br was incorporated into the chromosomes in place of ^13^C (Fig. 3). In this regard, ^13^C-rich chromatids were Br-poor and vice versa. In the carbon isotope ratio images, we could successfully define sister chromatid exchanges (SCEs), a testament to the high-resolution carbon ion-chromosomal images.

**Fig. 4:**
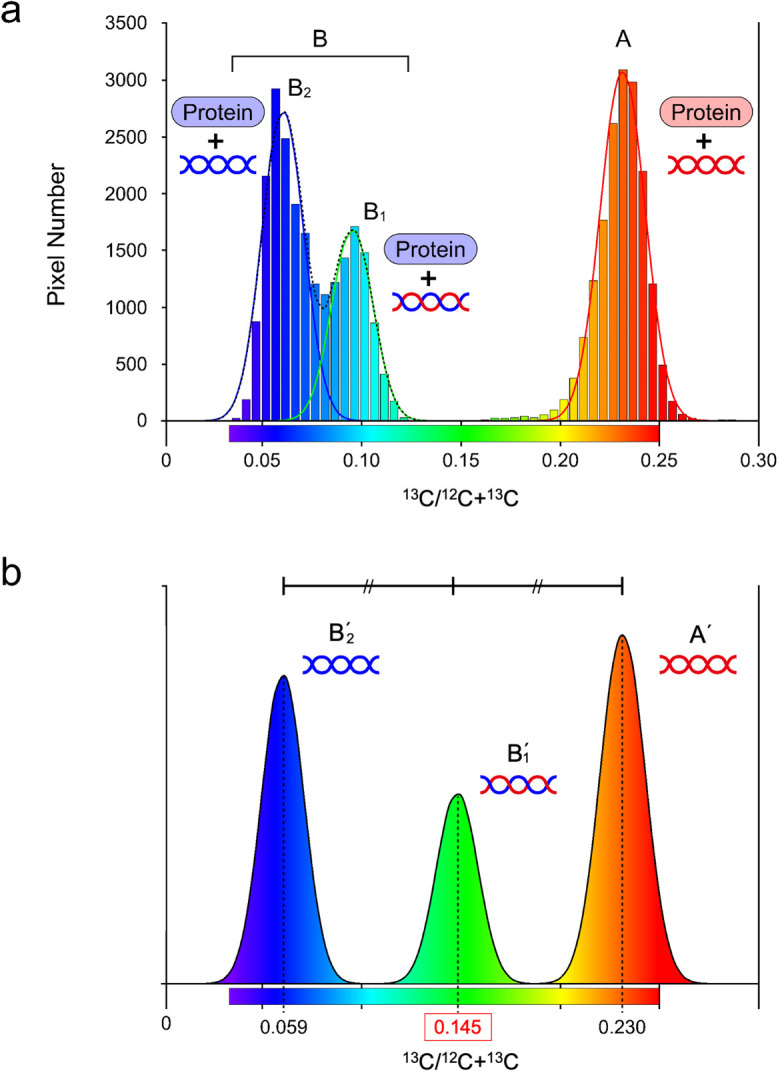
Histogram of carbon isotopes in chromosomes. (a) Carbon isotope ratio of chromatids analyzed in LIMAS imaging, shown in Fig. 2 (peak A) and Fig. 3 (peak B_1_ and B_2_). (b) Estimated carbon isotope ratio of DNA calculated from data in panel (a). The histone associated ^13^C in peaks B (B_1_ and B_2_) was considered as even and was subtracted from the total ^13^C in peak B_1_, resulting in peak B'_1_ with a carbon isotope ratio of 0.145. Each peak was fitted by the Gaussian curve (median±standard deviation). The red line in panel (a) indicates the Gaussian curve fitted to Peak A. Peak B_1_ and B_2_ (in Peak B) correspond to the ^13^C-poor and -rich chromatids, respectively.

From the carbon isotope images obtained in Fig. 2 and Fig. 3, we determined the carbon isotope ratios in individual chromatids (Fig. 4). Peak A (isotope ratio: 0.155–0.305) and peak B (0.030–0.130) correspond to the isotope ratio of Fig. 2. and Fig. 3C, respectively. The value of the valley between the two peaks (a yellow bin in Fig. S5a: 0.08–0.085) mostly corresponds to the region of sister chromatid cohesion (yellow pixels of Fig. S5b). Thus, histograms without the yellow bin were used for individual Gaussian curve fitting, using the least-squares method (Fig. 4a). Two Gaussian curves were fitted to Peak B_1_ (0.085–0.150) and Peak B_2_ (0–0.080) (green and blue lines of Fig. 4a, respectively). The composite of two Gaussian curves provides a good approximation of Peak B (dashed line of Fig. 4a).

The ratio of carbon in DNA and histone found in peak B_1_ can therefore be calculated. In this calculation, we consider that a nucleosome, the basic structural unit of a chromosome, consists of about 200 bp DNA and a pair of core and linker histones (Kornberg, 1974). Furthermore, in a human cell, the DNA consists of adenosine, thymidine, cytidine, and guanosine and contains 3,918 carbon atoms per nucleosome (Lander *et al.*, 2001). The core histone consists of 8 histone molecules (two molecules each of Histone H2A, H2B, H3, H4), while the linker histone consists of 1 histone molecule (Kornberg, 1974). Therefore, a histone contains 5,740 carbon atoms per nucleosome, calculated from the protein sequences (ensemble ver. ENSG00000277075.2, ENSG00000180596.7, ENSG00000197153.4, ENSG00000158406.4, ENSG00000189060.5). Therefore, the total number of carbons in a single nucleosome is 9,658. If the carbon isotope ratio of the histone in peak B_1_ is equivalent to that in peak B_2_, then the amount of carbon in DNA found in peak B_1_ can be calculated by subtracting the histone-associated carbon from peak B_1_, giving a DNA-associated carbon of 569. Then the carbon isotope ratio of DNA in peak B_1_ will be 0.145 (569/3,918) (Fig. 4b), corresponding to the midpoint of peak A' and B'_2_, indicates that the stable isotope labeling quantitatively visualized the semi-conservative replication of individual DNA strands in a single cell. These results also suggest that the histones in daughter chromosomes were renewed through cell division.

## Discussion

This study employed an analog-free, stable isotope labeling system in visualizing the chromosomes in Hela cells. We adopted stable isotope labeling with U-^13^C_6_-glucose to trace chromosomes through cell division. In cell culture systems, nucleotides (components of DNA) are synthesized from glucose in the medium via the pentose phosphate pathway and amino acid metabolism. Furthermore, glucose in the culture medium may also serve as a substrate for other biomolecules such as amino acids. The carbon isotope ratio of the culture medium used in this study was 0.238, and the detected carbon isotope ratio in the chromosomes was 0.230 (Fig. 2). This result confirms that most of the carbons in chromosomes were derived from glucose in the medium. The method has a 97% efficiency rate. Although proteins could also be labeled with ^13^C, the labeling efficiency for each molecule was not defined.

LIMAS imaging allowed us to quantify isotope ratios, which revealed the distinct distribution of isotopes between sister chromatids. The pairwise distribution of ^13^C-rich and ^13^C-poor regions within sister chromatids revealed by isotope imaging is consistent with the expected patterns of chromosome segregation after two rounds of the cell cycle. Moreover, the distribution of the ^13^C-poor region corresponded with that of the Br-rich region (Fig. 3). These results demonstrate the reliability of the stable isotope labeling system in tracing the dynamics of chromosome replication/segregation at subcellular resolution. The ^13^C-rich chromatids were composed of single-strand DNA synthesized in the U-^13^C_6_-Glucose-culture medium and single-strand DNA and proteins synthesized in the ^13^C-inverse pulse labeling-culture medium. The supply of nutrients in the ^13^C-inverse pulse labeling-culture medium, which had a natural carbon isotope ratio, and molecules in the parent cell, was essential for synthesizing the daughter cell components. Therefore, a carbon isotope ratio of 0.066 was expected in the second cell cycle from the fourfold dilution of the parent DNA and proteins (0.230) with the natural carbon isotope ratio (0.011) of the ^13^C-inverse pulse labeling-culture medium. The observed carbon isotope ratio of 0.059±0.010, in ^13^C-poor chromatids in the second cell cycle, was therefore consistent with the calculated value. The small deficit may be due to the metabolism of carbon sources during cell division. Thus, the carbon isotope ratio of peak B_2_ of Fig. 4 shows that the chromatids (DNA and proteins) were synthesized during the second cell cycle after medium replacement.

Several models have been proposed to explain histone incorporation into chromatids carrying epigenetic modifications during chromosome replication (Budhavarapu *et al.*, 2013; English *et al.*, 2017; Natsume *et al.*, 2007; Petryk *et al.*, 2018; Xie *et al.*, 2017; Yu *et al.*, 2018). Our results showed the renewal of histone molecules in daughter chromosomes, however, we could not see the inheritance process of histones, which carries epigenetic information. In other words, it is possible that epigenetic information is transmitted with parental histones thereafter histone molecules are refreshed in daughter cells.

Sister chromatids and the SCEs were distinguishable in LIMAS images. One example was the detection of SCEs. The strand-specific sequencing method demonstrated the effect of BrdU on SCEs (Van Wietmarschen and Lansdorp, 2016). Our imaging method will help the confirmation of SCE occurrence in nature. Another possible application of LIMAS bioimaging is tracing isotope-labeled organelles. The dynamics of organelles have been given less attention than DNA, and are just starting to be understood (Moore *et al.*, 2021). In this study, we labeled and chased chromosomes by using ^13^C-labeled glucose, which is a general carbon source for cellular molecules. This target molecule-independent labeling will be applicable for tracing other organelles such as mitochondria with appropriate sample preparation. Thus, the LIMAS analysis with the high spatial resolution will be a new analytical tool for various fields.

## References

[B1] Bajo, K., Itose, S., Matsuya, M., Ishihara, M., Uchino, K., Kudo, M., Sakaguchi, I., and Yurimoto, H. 2016. High spatial resolution imaging of helium isotope by TOF-SNMS. Surf. Interface Anal., 48: 1190–1193.

[B2] Bajo, K., Fujioka, O., Itose, S., Ishihara, M., Uchino, K., and Yurimoto, H. 2019. Electronic data acquisition and operational control system for time-of-flight sputtered neutral mass spectrometer. Surf. Interface Anal., 51: 35–39.

[B3] Bannigan, J. and Langman, J. 1979. The cellular effect of 5-bromodeoxyuridine on the mammalian embryo. J. Embryol. Exp. Morphol., 50: 123–135. 458350

[B4] Budhavarapu, V.N., Chavez, M., and Tyler, J.K. 2013. How is epigenetic information maintained through DNA replication? Epigenetics and Chromatin, 6: 1–7. 24225278 10.1186/1756-8935-6-32 PMC3852060

[B5] Ebata, S., Ishihara, M., Uchino, K., Itose, S., Matsuya, M., Kudo, M., Bajo, K., and Yurimoto, H. 2012. Development of laser ionization mass nanoscope (LIMAS). Surf. Interface Anal., 44: 635–640.

[B6] English, C.M., Maluf, N.K., Tripet, B., Churchill, M.E.A., and Tyler, J.K. 2017. ASF1 Binds to a Heterodimer of Histones H3 and H4: A Two-Step Mechanism for the Assembly of the H3–H4 Heterotetramer on DNA. Biochemistry, 176: 139–148. 10.1021/bi051333h PMC4445473 16229457

[B7] Gratzner, H.G., Leif, R.C., Ingram, D.J., and Castro, A. 1975. The use of antibody specific for bromodeoxyuridine for the immunofluorescent determination of DNA replication in single cells and chromosomes. Exp. Cell Res., 95: 88–94. 811484 10.1016/0014-4827(75)90612-6

[B8] Hamasaki, T., Matsumoto, T., Sakamoto, N., Shimahara, A., Kato, S., Yoshitake, A., Utsunomiya, A., Yurimoto, H., Gabazza, E.C., and Ohgi, T. 2013. Synthesis of ^18^O-labeled RNA for application to kinetic studies and imaging. Nucleic Acids Res., 41. 10.1093/nar/gkt344 PMC3695515 23632164

[B9] Kornberg, R.D. 1974. Chromatin Structure : A Repeating Unit of Histones and DNA. Science, 184: 868–871. 4825889 10.1126/science.184.4139.868

[B10] Kuga, Y., Sakamoto, N., and Yurimoto, H. 2014. Stable isotope cellular imaging reveals that both live and degenerating fungal pelotons transfer carbon and nitrogen to orchid protocorms. New Phytol. Trust, 202: 594–605. 10.1111/nph.12700 24494717

[B11] Lander, E.S., Linton, L.M., Birren, B., Nusbaum, C., Zody, M.C., Baldwin, J., Devon, K., Dewar, K., Doyle, M., Fitzhugh, W., et al. 2001. Initial sequencing and analysis of the human genome: International Human Genome Sequencing Consortium. Nature, 412: 565–566. 10.1038/35057062 11237011

[B12] Latt, S.A. 1973. Microfluorometric Detection of Deoxyribonucleic Acid Replication in Human Metaphase Chromosomes. Proc. Natl. Acad. Sci., 70: 3395–3399. 4128545 10.1073/pnas.70.12.3395 PMC427244

[B13] Lechene, C., Hillion, F., McMahon, G., Benson, D., Kleinfeld, A.M., Kampf, J.P., Distel, D., Luyten, Y., Bonventre, J., Hentschel, D., et al. 2006. High-resolution quantitative imaging of mammalian and bacterial cells using stable isotope mass spectrometry. J. Biol., 5: 20. 17010211 10.1186/jbiol42 PMC1781526

[B14] Lehner, B., Sandner, B., Marschallinger, J., Lehner, C., Furtner, T., Couillard-Despres, S., Rivera, F.J., Brockhoff, G., Bauer, H.C., Weidner, N., et al. 2011. The dark side of BrdU in neural stem cell biology: Detrimental effects on cell cycle, differentiation and survival. Cell Tissue Res., 345: 313–328. 21837406 10.1007/s00441-011-1213-7

[B15] Levi-Setti, R. and Le Beau, M. 1992. Cytogenetic applications of high resolution secondary ion imaging microanalysis: detection and mapping of tracer isotopes in human chromosomes. Biol. Cell, 74: 51–58. 1511248 10.1016/0248-4900(92)90008-o

[B31] Moore, A.S., Coscia, S.M., Simpson, C.L., Ortega, F.E., Wait, E.C., Heddleston, J.M., Nirschl, J.J., Obara, C.J., Guedes-Dias, P., Boecker, C.A., et al. 2021. Actin cables and comet tails organize mitochondrial networks in mitosis. 10.1038/s41586-021-03309-5 PMC7990722 33658713

[B16] Nagata, K., Bajo, K., Itose, S., Matsuya, M., Ishihara, M., Uchino, K., and Yurimoto, H. 2019. Aberration-corrected focused ion beam for time-of-flight secondary neutral mass spectrometry. Appl. Phys. Express, 12: 085005.

[B17] Natsume, R., Eitoku, M., Akai, Y., Sano, N., Horikoshi, M., and Senda, T. 2007. Structure and function of the histone chaperone CIA/ASF1 complexed with histones H3 and H4. Nature, 446: 338–341. 17293877 10.1038/nature05613

[B18] Perry, P. and Wolff, S. 1974. New Giemsa method for the differential staining of sister chromatids. Nature, 251: 156–158. 4138930 10.1038/251156a0

[B19] Petryk, N., Dalby, M., Wenger, A., Stromme, C.B., Strandsby, A., Andersson, R., and Groth, A. 2018. MCM2 promotes symmetric inheritance of modified histones during DNA replication. Science (80-. ), 361: 1389–1392. 10.1126/science.aau0294 30115746

[B20] Pinkel, D., Thompson, L.H., Gray, J.W., and Vanderiaan, M. 1985. Measurement of Sister Chromatid Exchanges at Very Low Bromodeoxyuridine Substitution Levels Using a Monoclonal Antibody in Chinese Hamster Ovary Cells. Cancer Res., 45: 5795–5798. 4053051

[B21] Ross, H.H., Levkoff, L.H., Marshall, G.P., Caldeira, M., Steindler, D.A., Reynolds, B.A., and Laywell, E.D. 2008. Bromodeoxyuridine Induces Senescence in Neural Stem and Progenitor Cells. Stem Cells, 26: 3218–3227. 18802036 10.1634/stemcells.2008-0299 PMC4541772

[B22] Solary, E., Bertrand, R., Jenkins, J., and Pommier, Y. 1992. Radiolabeling of DNA can induce its fragmentation in HL-60 human promyelocytic leukemic cells. Exp. Cell Res., 203: 495–498. 1459209 10.1016/0014-4827(92)90027-6

[B23] Steinhauser, M.L., Bailey, A.P., Senyo, S.E., Guillermier, C., Perlstein, T.S., Gould, A.P., Lee, R.T., and Lechene, C.P. 2012. Multi-isotope imaging mass spectrometry quantifies stem cell division and metabolism. Nature, 481: 516–519. 22246326 10.1038/nature10734 PMC3267887

[B24] Taupin, P. 2007. BrdU immunohistochemistry for studying adult neurogenesis: Paradigms, pitfalls, limitations, and validation. Brain Res. Rev., 53: 198–214. 17020783 10.1016/j.brainresrev.2006.08.002

[B25] Taylor, J.H., Woods, P.S., and Hughes, W.L. 1957. The Organization and Duplication of Chromosomes As Revealed By Autoradiographic Studies Using Tritium-Labeled Thymidinee. Proc. Natl. Acad. Sci., 43: 122–128. 16589984 10.1073/pnas.43.1.122 PMC528395

[B26] Tonotani, A., Bajo, K., Itose, S., Ishihara, M., Uchino, K., and Yurimoto, H. 2016. Evaluation of multi-turn time-of-flight mass spectrum of laser ionization mass nanoscope. Surf. Interface Anal., 48: 1122–1126.

[B27] Van Wietmarschen, N. and Lansdorp, P.M. 2016. Bromodeoxyuridine does not contribute to sister chromatid exchange events in normal or Bloom syndrome cells. Nucleic Acids Res., 44: 6787–6793. 27185886 10.1093/nar/gkw422 PMC5001594

[B28] Xie, J., Wooten, M., Tran, V., and Chen, X. 2017. Breaking Symmetry—Asymmetric Histone Inheritance in Stem Cells. Trends Cell Biol., 27: 527–540. 28268050 10.1016/j.tcb.2017.02.001 PMC5476491

[B29] Yu, C., Gan, H., Serra-Cardona, A., Zhang, L., Gan, S., Sharma, S., Johansson, E., Chabes, A., Xu, R.M., and Zhang, Z. 2018. A mechanism for preventing asymmetric histone segregation onto replicating DNA strands. Science (80-. )., 361: 1386–1389. 10.1126/science.aat8849 PMC6597248 30115745

[B30] Yurimoto, H., Bajo, K., Sakaguchi, I., Suzuki, T.T., Jurewicz, A.J.G., Itose, S., Uchino, K., and Ishihara, M. 2016. Quantitative analysis of helium by post-ionization method using femtosecond laser technique. Surf. Interface Anal., 48: 1181–1184.

