# Supplementary material for: Visualization of DNA Replication in Single Chromosome by Stable Isotope Labeling
Source: Cell Struct Funct. 2021 Sep 25;46(2):95–101. doi: 10.1247/csf.21011 (PMC10511050; doi:10.1247/csf.21011)
Supplement: Supplementary file 1 — Fig. S1 [file csf_46_21011_1.pdf]

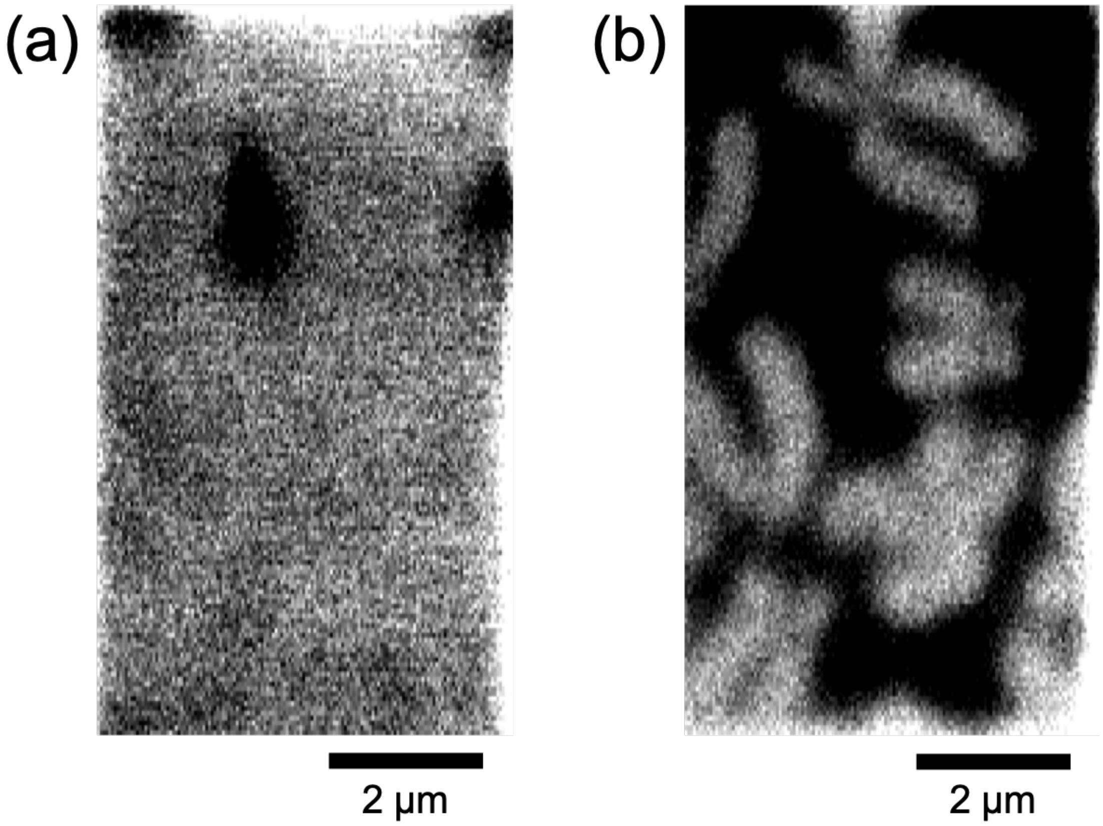

Fig. S1. ^{12}C ion images of chromosomes before and after pre-sputtering. (a) Chromosomes covered by cell components before pre-sputtering. (b) Visible chromosomes after sputtering of cell components.
