# Supplementary figures and images for: Visualization of DNA Replication in Single Chromosome by Stable Isotope Labeling
Source: Cell Struct Funct. 2021 Sep 25;46(2):95–101. doi: 10.1247/csf.21011 (PMC10511050; doi:10.1247/csf.21011)

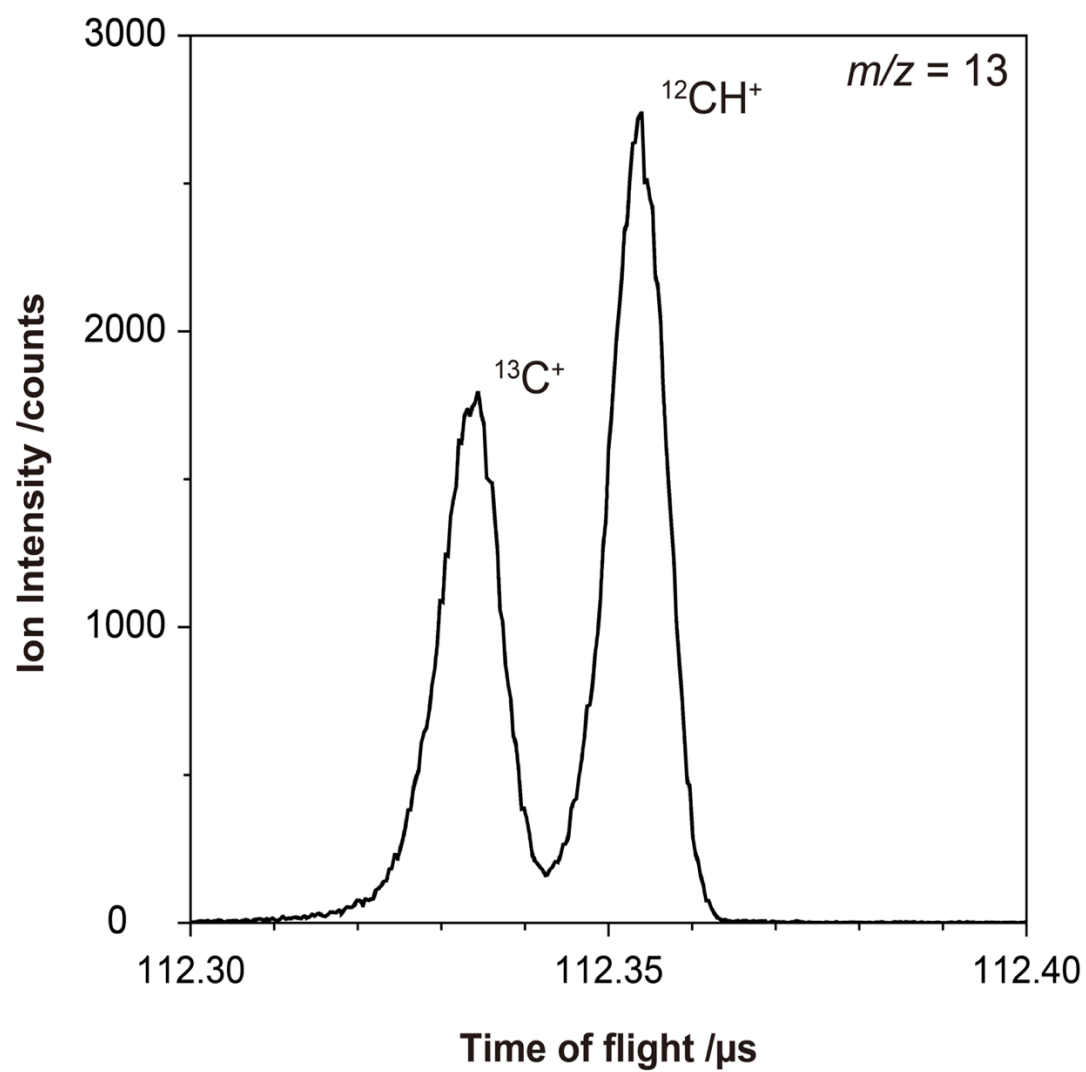

**Fig. S2.** Mass spectrum against time of flight (TOF). The TOF of 112.3–112.4  $\mu\text{s}$  corresponds to  $m/z = 13$ .

Supplement: Supplementary file 2 — Fig. S2 [file csf_46_21011_2.pdf]
