# Supplementary material for: Visualization of DNA Replication in Single Chromosome by Stable Isotope Labeling
Source: Cell Struct Funct. 2021 Sep 25;46(2):95–101. doi: 10.1247/csf.21011 (PMC10511050; doi:10.1247/csf.21011)
Supplement: Supplementary file 3 — Fig. S3 [file csf_46_21011_3.pdf]

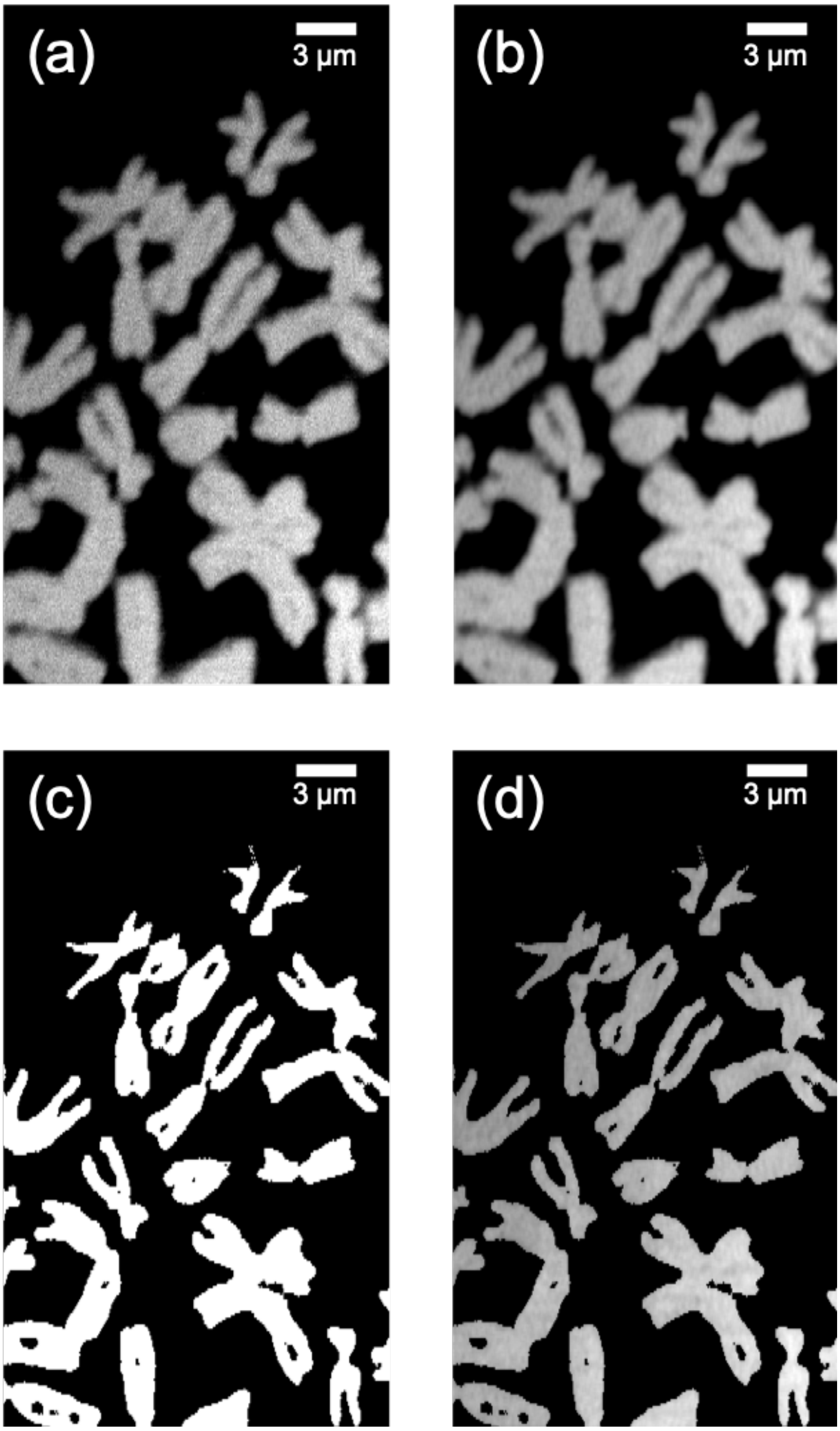

Fig. S3. Carbon (^{12}C + ^{13}C) images of the area captured in the red square of Fig. 3B. (a) Raw image. (b) 3 × 3 smoothing image. (c) Mask of chromosomes made by ^{28}Si image. (d) 3 × 3 smoothing image of chromosomes.
