# Supplementary material for: Visualization of DNA Replication in Single Chromosome by Stable Isotope Labeling
Source: Cell Struct Funct. 2021 Sep 25;46(2):95–101. doi: 10.1247/csf.21011 (PMC10511050; doi:10.1247/csf.21011)
Supplement: Supplementary file 4 — Fig. S4 [file csf_46_21011_4.pdf]

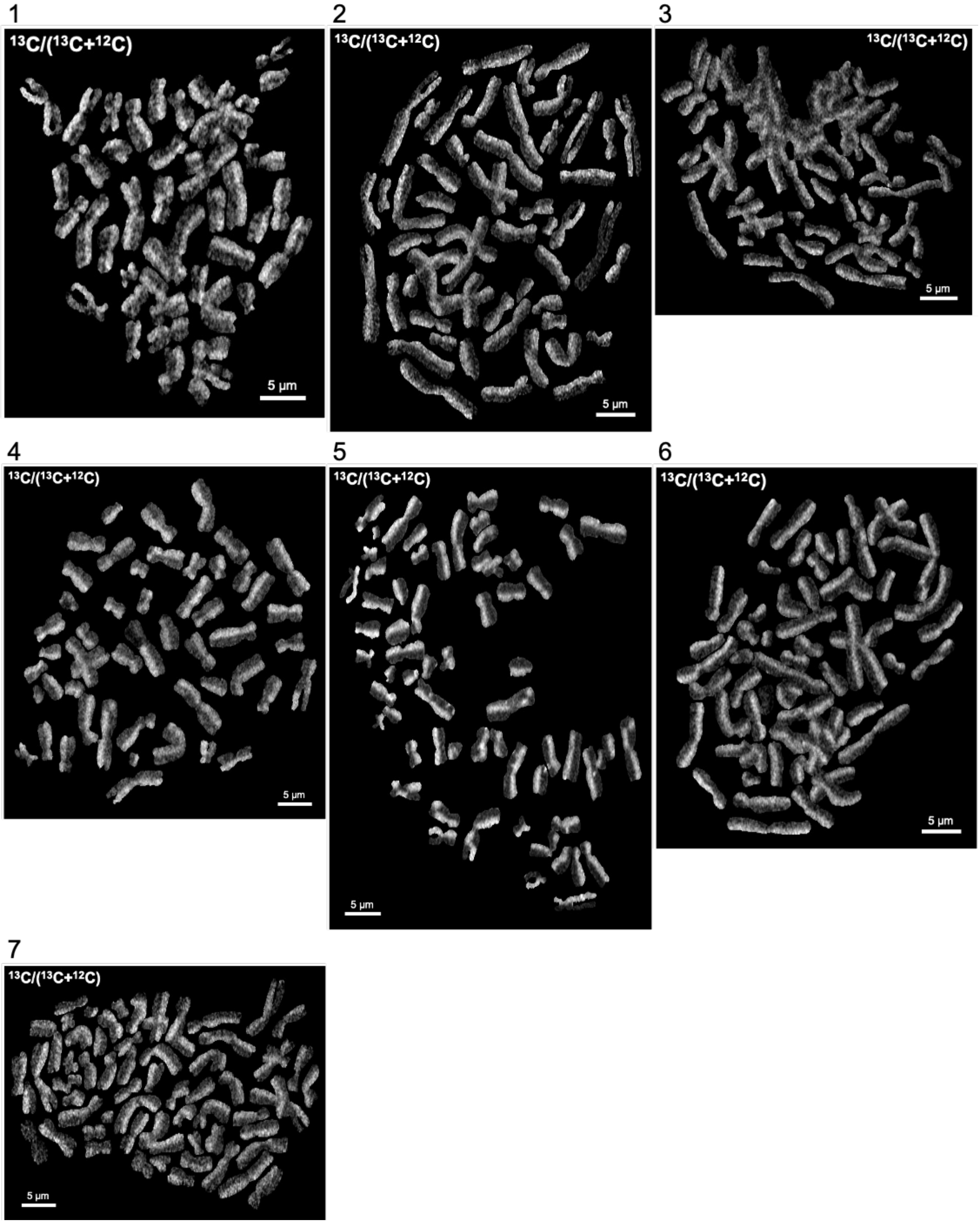

Fig. S4. ^{13}C distributions in chromosomes, which were labeled under the ^{13}C-inverse pulse labeling.
