# Supplementary material for: Visualization of DNA Replication in Single Chromosome by Stable Isotope Labeling
Source: Cell Struct Funct. 2021 Sep 25;46(2):95–101. doi: 10.1247/csf.21011 (PMC10511050; doi:10.1247/csf.21011)
Supplement: Supplementary file 5 — Fig. S5 [file csf_46_21011_5.pdf]

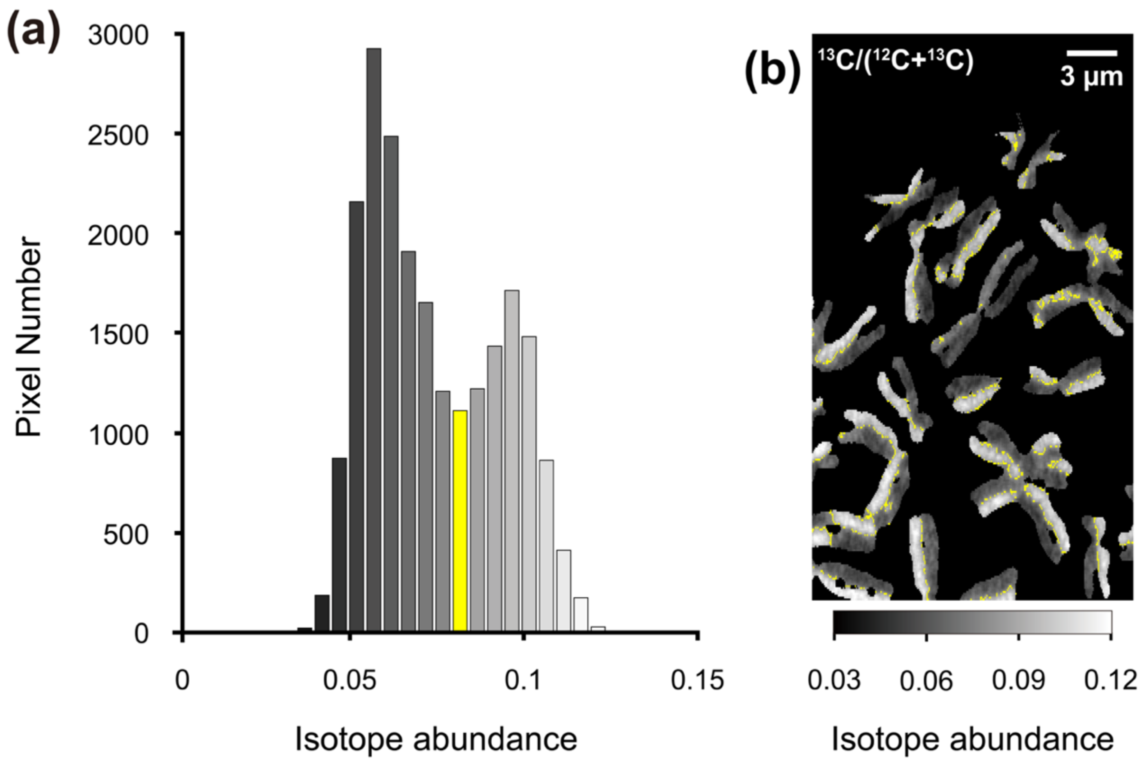

Fig. S5. Corresponding position in isotope image for intermediate bin of peak B of Fig. 4. (a) The intermediate bin highlighted in yellow. (b) Corresponding pixels of the yellow-colored bin.
